# Effectiveness of XBB.1.5 Monovalent COVID‐19 Vaccines During a Period of XBB.1.5 Dominance in EU/EEA Countries, October to November 2023: A VEBIS‐EHR Network Study

**DOI:** 10.1111/irv.13292

**Published:** 2024-04-23

**Authors:** Susana Monge, James Humphreys, Nathalie Nicolay, Toon Braeye, Izaak Van Evercooren, Christian Holm Hansen, Hanne‐Dorthe Emborg, Chiara Sacco, Alberto Mateo‐Urdiales, Jesús Castilla, Iván Martínez‐Baz, Brechje de Gier, Susan Hahné, Hinta Meijerink, Anja Bråthen Kristoffersen, Ausenda Machado, Patricia Soares, Anthony Nardone, Sabrina Bacci, Esther Kissling, Baltazar Nunes, VEBIS‐EHR Working Group

**Affiliations:** ^1^ Department of Communicable Diseases, National Centre of Epidemiology Institute of Health Carlos III Madrid Spain; ^2^ CIBER on Infectious Diseases Madrid Spain; ^3^ Epiconcept Paris France; ^4^ Vaccine Preventable Diseases and Immunisation European Centre for Disease Prevention and Control (ECDC) Solna Sweden; ^5^ Sciensano Elsene Belgium; ^6^ Department of Infectious Disease Epidemiology and Prevention Statens Serum Institut Copenhagen Denmark; ^7^ Infectious Diseases Department Istituto Superiore di Sanità Rome Italy; ^8^ European Programme on Intervention Epidemiology Training (EPIET) European Centre for Disease Prevention and Control Stockholm Sweden; ^9^ Instituto de Salud Pública de Navarra – IdiSNA Pamplona Spain; ^10^ CIBER on Epidemiology and Public Health Madrid Spain; ^11^ Center for Infectious Disease Control National Institute for Public Health and the Environment (RIVM) Bilthoven The Netherlands; ^12^ Norwegian Institute of Public Health (NIPH) Oslo Norway; ^13^ Departamento de Epidemiologia Instituto Nacional de Saúde Doutor Ricardo Jorge Lisboa Portugal

**Keywords:** cohort design, COVID‐19, electronic health records, hospitalisation, multicountry study, SARS‐CoV‐2, vaccine effectiveness

## Abstract

Using a common protocol across seven countries in the European Union/European Economic Area, we estimated XBB.1.5 monovalent vaccine effectiveness (VE) against COVID‐19 hospitalisation and death in booster‐eligible ≥ 65‐year‐olds, during October–November 2023. We linked electronic records to construct retrospective cohorts and used Cox models to estimate adjusted hazard ratios and derive VE. VE for COVID‐19 hospitalisation and death was, respectively, 67% (95%CI: 58–74) and 67% (95%CI: 42–81) in 65‐ to 79‐year‐olds and 66% (95%CI: 57–73) and 72% (95%CI: 51–85) in ≥ 80‐year‐olds. Results indicate that periodic vaccination of individuals ≥ 65 years has an ongoing benefit and support current vaccination strategies in the EU/EEA.

## 1 Introduction

COVID‐19 vaccination in European Union/European Economic Area (EU/EEA) countries has been rolled out as a 2023 autumnal campaign targeting vulnerable populations, mostly in individuals aged ≥ 60 or ≥ 65 years and those with comorbidities. Periodic vaccination in target groups has been implemented to maintain protection against severe COVID‐19 outcomes due to waning of immunity and rapid decrease in vaccine effectiveness (VE) seen in recent vaccination campaigns [1, 2, 3, 4]. Adapted vaccines have been approved to match SARS‐CoV‐2 circulating variants and increase protection in periods of dominance of BA.4 or BA.5 and XBB.1.5 and have been used in the EU/EEA [1, 2, 3, 4, 5, 6, 7, 8, 9], although a new Omicron subvariant, BA.2.86, became dominant in EU/EEA countries around late November and early December 2023 [10].

Our objective was to estimate VE against COVID‐19 hospitalisation and death of XBB.1.5 monovalent vaccines administered as 2023 autumnal booster dose, during the period where there was generally a match between the vaccine composition and the dominant SARS‐CoV‐2 variant (October and November 2023).

## 2 Study Setting and Methods

Since October 2021, a multicountry COVID‐19 VE study using electronic health records (EHR) has been ongoing in EU/EEA countries under the Vaccine Effectiveness Burden and Impact Studies (VEBIS) project, funded by the European Centre of Disease Prevention and Control (ECDC). As of January 2024, seven countries participate in the VEBIS‐EHR network: Belgium, Denmark, Italy, Spain (Navarra), Norway, Portugal and the Netherlands.

Detailed methods and results have been previously published [11, 12, 13, 14]. Briefly, using a common protocol, retrospective fixed cohorts were constructed in participating countries using deterministic linkage of population and health databases. For the monitoring of the 2023/2024 season [12], we included individuals eligible to receive the autumnal booster vaccination at the beginning of the campaign (Table 1), specifically, those 65 years or older, with at least complete primary vaccination and who, in the last 90 days, had no vaccine dose administered and no documented SARS‐CoV‐2 infection. Individuals contributed person‐days to the ‘non‐exposed’ to the 2023 autumnal booster since the beginning of the study and up to the date of administration of the autumnal booster or the end of follow‐up. Individuals' person‐days started contributing to the 2023 autumnal booster ‘exposed’ group 14 days after receiving the booster. The first 14 days after receipt of the booster were dropped from the analysis, and consistently, the study period started 14 days after the start of the vaccination campaign in each country. Follow‐up in both groups ceased at the date of the outcome, date of death for any cause or end of the study period (25 November 2023).

**TABLE 1:** Start of the COVID‐19 2023 autumnal vaccination campaign in participating countries.

Study site	Date of start of the vaccination campaign
Belgium	11 September 2023
Denmark	1 October 2023
Italy	27 September 2023^a^
Navarra (Spain)	16 October 2023
The Netherlands	2 October 2023
Norway	1 September 2023
Portugal	29 September 2023

^a^ Day of publication of the Ministerial Circular Law with the recommendations on which vaccines to use and to who it should be offered. The actual day of start varied across regions, with most starting the first week of October.

Hospitalisation due to COVID‐19 was defined as a hospital admission due to a severe acute respiratory infection with a SARS‐CoV‐2 positive test from 14 days before to 1 day after admission or with COVID‐19 as the main diagnosis in admission or discharge records, except in the Netherlands, where admissions with a positive SARS‐CoV‐2 test and missing or unknown reason for admission (about 50% of all admissions with a positive SARS‐CoV‐2 test) were also included.COVID‐19‐related death was defined as death for which COVID‐19 was recorded as the main cause (even with no positive SARS‐CoV‐2 test) or, if the cause was not available, laboratory‐confirmed SARS‐CoV‐2 infection with death in the 30 days after the positive test or symptom onset.

Cox regression with calendar time as the underlying scale was used to estimate vaccine hazard ratios and derived VE [HR; VE = (1 − HR) × 100] and 95% confidence intervals (95%CI), adjusting by 5‐year age‐groups, sex, region in the country, comorbidities and previous number of vaccine booster doses. Site‐specific estimates were pooled using a random‐effects meta‐analysis, given expected heterogeneity from study design and disease epidemiology. To estimate heterogeneity, we used the I2 index [15]. A fixed‐effects model was used as secondary analysis.

## 3 Results

In the group aged 65–79 years, 23.3 and 1.9 million person‐days were included in the non‐exposed and exposed groups, respectively; 5775 hospitalisations due to COVID‐19 and 386 COVID‐related deaths were included in the non‐exposed group versus 207 and 16 in the exposed. In the group aged ≥ 80 years, 9.5 and 1.0 million person‐days were included in the nonexposed and exposed groups, respectively; 8497 hospitalisations due to COVID‐19 and 1200 COVID‐related deaths were included in the nonexposed group versus 312 and 34 in the exposed. Individuals in the exposed group had more frequent comorbidities compared to the nonexposed (Table 2). As of November 2023, the autumnal boosters were mostly Pfizer mRNA XBB.1.5 monovalent vaccines (98%).

**TABLE 2:** Descriptive characteristics by vaccination status, VEBIS‐EHR study, October to November 2023.

Variable		Not vaccinated		Vaccinated	
		Count	% of cohort total	Count	% of cohort total
Sex	Female	9,712,250	55%	2,378,148	53%
	Male	7,901,263	45%	2,118,470	47%
	Missing	22	0%	< 5	—
Comorbidities	High‐risk comorbidities/immunocompromising	479,967	3%	393,205	9%
	Medium‐risk comorbidities/non‐immunocompromising	6,398,375	36%	2,261,177	50%
	No comorbidity	10,708,009	61%	1,829,422	41%
	Missing	27,184	0%	12,815	0%
Number of previous vaccine boosters	0	1,745,023	10%	16,981	0%
	1	8,386,032	48%	226,135	5%
	2	6,301,163	36%	2,837,619	63%
	3	1,177,481	7%	1,413,806	31%
	4	2472	0%	16	0%
	5	39	0%	—	0%
	Missing	1325	0%	2062	0%
Country of birth	Native	13,243,192	75%	1,397,411	31%
	Non‐native	508,094	3%	39,082	1%
	Missing	3,862,249	22%	3,060,126	68%
Nationality	National	1,726,175	10%	1,121,558	25%
	Nonnational	330,884	2%	57,029	1%
	Missing	15,556,476	88%	3,318,032	74%
Vaccine product	Pfizer (monovalent)	—	—	61,332	1%
	Moderna (monovalent)	—	—	60	0%
	Pfizer (bivalent original/BA.1)	—	—	4006	0%
	Moderna (bivalent original/BA.1)	—	—	10	0%
	Pfizer (bivalent original/BA.4/BA.5)	—	—	10,914	0%
	Moderna (bivalent original/BA.4/BA.5)	—	—	116	0%
	Pfizer (XBB.1.5)	—	—	4,370,556	97%
	Moderna (XBB.1.5)	—	—	49,624	1%
	Novavax	—	—	—	0%
	Other (all other types, including AZ)	—	—	< 5	—
	Missing	—	—	—	0%

**FIGURE 1:**
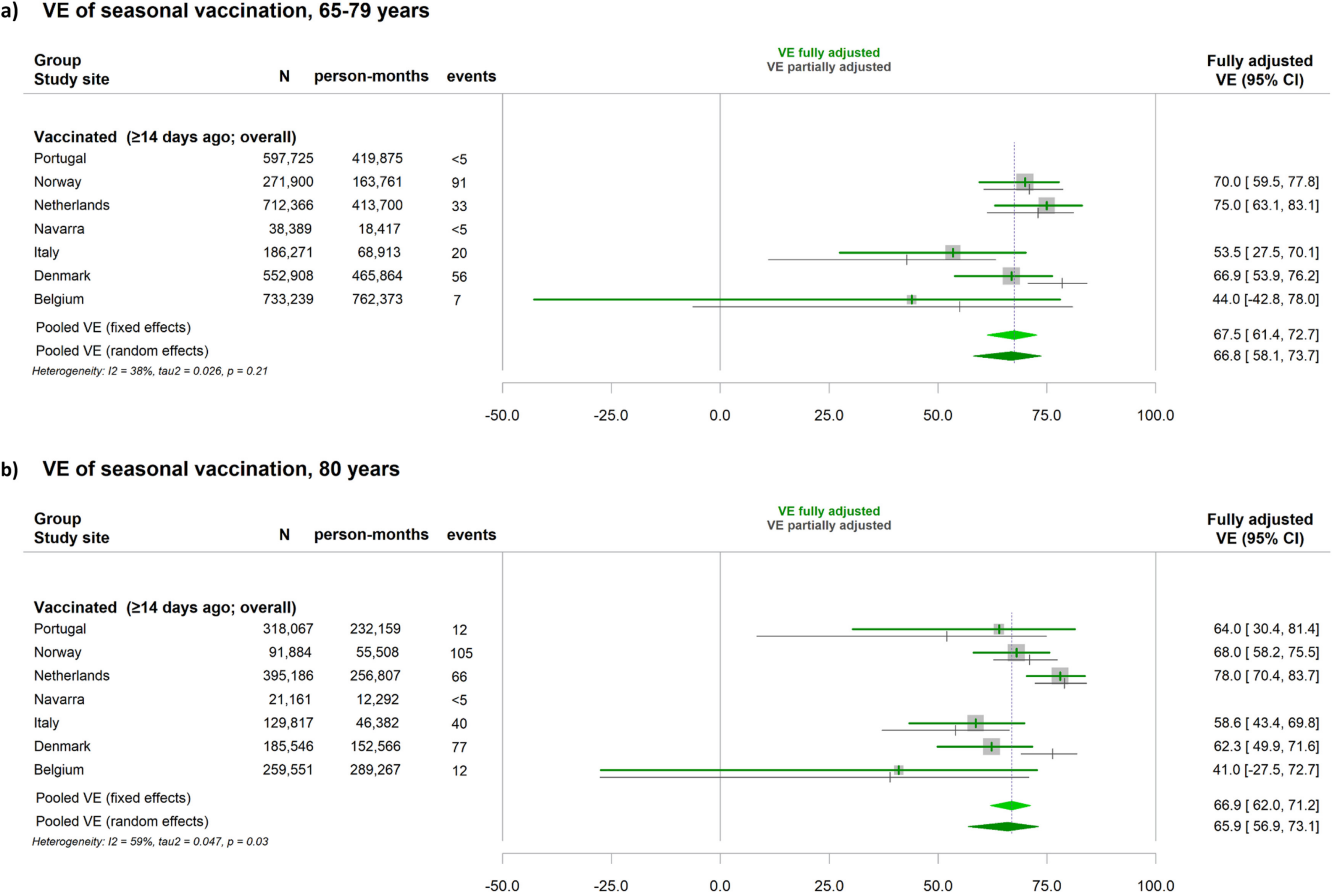
Vaccine effectiveness (VE) of 2023 autumnal monovalent XBB.1.5 vaccination against hospitalisation due to COVID‐19 in seven European VEBIS‐EHR study sites and pooled estimate using either fixed‐effects or random‐effects meta‐analysis. (a) In the group aged 65–79 years; (b) in the group aged 80 years and older, October to November 2023.

**FIGURE 2:**
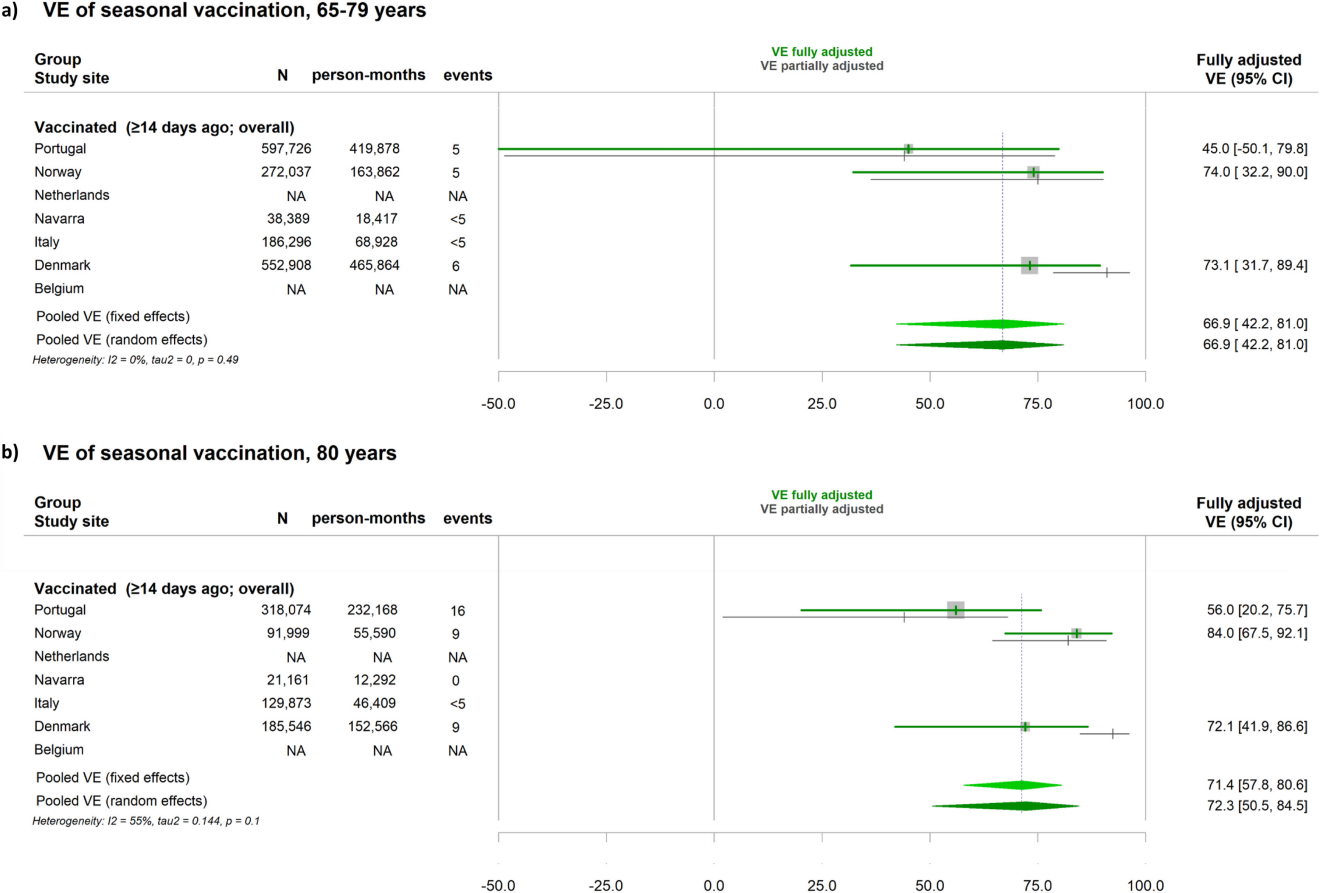
Vaccine effectiveness (VE) of 2023 autumnal monovalent XBB.1.5 vaccination against COVID‐19 related death in seven European VEBIS‐EHR study sites and pooled estimate using either fixed‐effects or random‐effects meta‐analysis. (a) In the group aged 65–79 years; (b) in the group aged 80 years and older, October to November 2023.

Site‐specific and pooled VE estimates are shown in Figure 1 for hospitalisation due to COVID‐19 and in Figure 2 for COVID‐19–related death. There was certain heterogeneity across study sites, which was lower in the group 65–79, for both hospitalisation and death (38% and 0%, respectively), than for those aged ≥ 80 years (59% and 55%). Random‐effects pooled VE against hospitalisation due to COVID‐19 or COVID‐19–related death were, respectively, 67% (95%CI: 58–74) and 67% (95%CI: 42–81) in those aged 65–79 years and 66% (95%CI: 57–73) and 72% (95%CI: 51–85) in the group aged ≥80 years, though VE against death was based on three study sites.

## 4 Discussion

Our estimates of VE with XBB.1.5 vaccines are comparable to others in this same period, when there was a match between the vaccine composition and the dominant SARS‐CoV‐2 variant [6]. Interestingly, because the VEBIS EHR monitoring network has been ongoing since October 2021, we are able to compare with the VE from the previous vaccination campaign, despite some changes in the methods and in the participating countries that have occurred in the network [11, 12, 13]. While, for previous boosters, we estimated VE according to the number of vaccine doses, for this autumnal dose, the network has transitioned to a seasonal approach, similar to the influenza VE monitoring framework [12]. Nevertheless, the VE as of November 2023 is comparable to the one estimated within this network in the 2022/23 between 1 October and 25 November, 2022, when estimated VE against hospitalisation due to COVID‐19 was, in those aged 65–79 years, 77% (95%CI: 66–83) for the second booster and, in the group aged ≥ 80 years, 76% (95%CI: 65–83) for the second booster and 71% (95%CI: 60–79) for the third booster [4]. Effectiveness was lower to the one estimated with a first booster dose in the autumn of 2021 [4]. Higher background immunity in the population and increased probability of selection of individuals with comorbidities in more recent vaccination campaigns may account, at least partially, for these differences.

The similarity between the current VE estimates and the ones from previous years is in agreement with other results from the VEBIS‐EHR network, which indicate that time since vaccination seems more relevant for protection than the number of previous COVID‐19 vaccine boosters [4, 14]. This supports the recommendation of having offered a new dose of COVID‐19 vaccines before a period of expected high burden of disease, such as that of the respiratory winter season, regardless of total number of previous vaccine boosters received. Also, our results confirm that periodic vaccination of individuals aged 65 years or older has an ongoing benefit, and support the current vaccination strategies in the EU/EEA countries. Ongoing monitoring of COVID‐19 VE in the coming months is needed to determine if the advent of the BA.2.86 variant has impacted the protection conferred by the XBB.1.5 boosters and the duration of protection by time since vaccination. Stable multicountry networks provide unique opportunities for ongoing monitoring of COVID‐19 vaccines VE and are key to inform public health recommendations.

## Author Contributions

**Susana Monge:** Conceptualization; Investigation; Funding acquisition; Writing – original draft; Methodology; Visualization; Supervision. **James Humphreys:** Conceptualization; Writing – review and editing; Validation; Methodology; Visualization; Formal analysis; Data curation. **Nathalie Nicolay:** Conceptualization; Investigation; Funding acquisition; Writing – review and editing; Methodology; Project administration; Supervision; Resources. **Toon Braeye:** Writing – review and editing; Formal analysis; Methodology; Data curation; Validation. **Izaak Van Evercooren:** Methodology; Validation; Writing – review and editing; Formal analysis; Data curation. **Christian Holm Hansen:** Methodology; Validation; Writing – review and editing; Formal analysis; Data curation. **Hanne‐Dorthe Emborg:** Methodology; Validation; Writing – review and editing; Formal analysis; Data curation. **Chiara Sacco:** Methodology; Validation; Writing – review and editing; Formal analysis; Data curation. **Alberto Mateo‐Urdiales:** Methodology; Validation; Writing – review and editing; Formal analysis; Data curation. **Jesús Castilla:** Methodology; Validation; Writing – review and editing; Formal analysis; Data curation. **Iván Martínez‐Baz:** Methodology; Validation; Writing – review and editing; Formal analysis; Data curation. **Brechje de Gier:** Methodology; Validation; Writing – review and editing; Formal analysis; Data curation. **Susan Hahné:** Methodology; validation; Writing – review and editing; Formal analysis; Data curation. **Hinta Meijerink:** Methodology; Validation; Writing – review and editing; Formal analysis; Data curation. **Anja Bråthen Kristoffersen:** Methodology; Validation; Writing – review and editing; Formal analysis; Data curation. **Ausenda Machado:** Methodology; Validation; Writing – review and editing; Formal analysis; Data curation. **Patricia Soares:** Methodology; Validation; Writing – review and editing; Formal analysis; Data curation. **Anthony Nardone:** Conceptualization; Funding acquisition; Writing – review and editing; Methodology; Project administration; Supervision. **Sabrina Bacci:** Project administration; Methodology; Writing – review and editing; Conceptualization; Funding acquisition; Supervision; Resources. **Esther Kissling:** Conceptualization; Investigation; Methodology; Writing – review and editing; Project administration; Supervision. **Baltazar Nunes:** Supervision; Project administration; Methodology; Conceptualization; Investigation; Writing – original draft; Visualization. **VEBIS‐EHR working group:** Data curation; Formal analysis; Software.

## Conflicts of Interest

The authors declare no conflicts of interest.

### Peer Review

The peer review history for this article is available at https://www.webofscience.com/api/gateway/wos/peer‐review/10.1111/irv.13292.

## Data Availability

Authors cannot share the data used for this study, which should be requested to the data owner institutions following their respective procedures.
